# The Impact of Servant Leadership on Proactive Service Behavior: a Moderated Mediation Model

**DOI:** 10.1186/s40359-024-01669-x

**Published:** 2024-03-28

**Authors:** Yinan Zhang, Yue Yin, Weilin Su

**Affiliations:** School of Literature, Capital Normal University, 83 Xisanhuan North Road, 100048 Beijing, China

**Keywords:** Civil servant, Proactive service behavior, Servant leadership, Public service motivation, Role identity

## Abstract

As the implementers of government policies, junior civil servants bear the responsibility of providing services to the public. Whether they can put themselves in the people’s shoes and show more active service consciousness directly reflects the government’s management ability and the realization of service-oriented government goals. Although proactive service behavior has been studied, it has not been brought into the field of government administration. Hence, from the perspective of servant leadership, this study attempts to introduce proactive service behavior (PSB) into the field of government administration, and discusses the promotion strategies of junior civil servants’ PSB in China. Through the statistical analysis of 416 junior civil servants collected in the two stages, this study verifies that servant leadership has a significant positive impact on public service motivation and PSB of junior civil servants. Public service motivation (PSM) can partially mediate the promotion effect of servant leadership on junior civil servants’ PSB. Role identity can positively moderate the relationship between servant leadership and junior civil servants’ PSM, and then affect their PSB. Therefore, this study suggests that leading cadres should integrate servant leadership into daily life, take the lead in providing quality services to their subordinates, and then inspire more proactive service to the masses.

## Introduction

Since 2004, important meetings of the Communist Party of China and the Government Work Reports have repeatedly made explicit requirements for promoting the construction of “service-oriented government” [1]. The Sixth Plenary Session of the 19th Central Committee of the Communist Party of China further summarized “putting the people first” as one of the ten valuable historical experiences over the past century since its foundation [2], which has also put forward higher requirements on how to build a service-oriented government adhering to people-oriented value [3]. Meanwhile, the concept of service-oriented government needs to be embodied in the daily practice of government departments and their employees [4]. As the implementers faced directly with the people and various policies and regulations developed by the Party and government [5], whether junior civil servants can perform their administrative responsibilities conscientiously in the daily practice [6], put themselves in the shoes of the masses and even demonstrate more PSB beyond the call of their duty, fundamentally determines the accomplishment of ambitious goal of building a people-satisfied and service-oriented government [7, 8]. Hence, it is of great practical significance to explore the strategies to improve PSB of junior civil servants in China. Due to the administrative divisions of China, which are mainly divided into three levels of organizational units, such as provinces, cities and counties, the junior civil servants in this study refers to those working at the county level and below.

The concept of PSB comes from the field of Service Management, and it refers to service behavior that the organization members perform responsibility conscientiously beyond self-role and organizational requirements, with the characteristics to be spontaneous, forward-looking and consistent [9]. According to the existing researches, previous studies have examined its predictors from different levels. For example, individual elements such as emotional labor [10], proactive personality [11], self-efficacy [9], and external factors like work climate [12], HR practices [13], leadership styles [14] have been discussed before, all exerting an important impact on PSB. When it comes to the influence of leadership styles on PSB, concepts like transformational leadership [15], humorous leadership [16], authentic leadership [17], servant leadership [18] gradually seize the focus of the researchers. As a leadership style of putting serving others as its core ideology [19], servant leadership emphasizes the priority of others’ demands, wishes and benefits [20, 21], then constantly obtaining trust of subordinates and probably influencing individual’s PSB. Russell (2001) emphasizes three attributes of servant leadership, trust, appreciation of others and empowerment, which can conduce to better subordinates’ behaviors [22]. Brewer (2010) mentions that servant leadership helps build intimacy that raises performance to a higher standard, improve job satisfaction and make commitment to the growth of people [23]. Servant leadership had been practiced and advocated in some of the best companies to work for in America, on the basis of the *Fortune* survey [24], which demonstrates the universal value in the field of management. However, to the best of our knowledge, there is no study that directly discusses the influence of servant leadership on PSB of junior civil servants under the circumstance of Chinese government departments. Only Miao and his colleagues have explored how servant leadership affects trust and organizational commitment in the Chinese public sector [25]. Therefore, this study attempts to take Chinese junior civil servants as the research object and explore the influence of servant leadership on their PSB and its mechanism.

Public Service Motivation (PSM) is an important factor of service capability of civil servants. PSM is defined as an individual’ s predisposition to respond to motives grounded primarily or uniquely in public institutions, measured by “attraction to policy making, commitment to the public interest, compassion and self-sacrifice” [26]. Essentially the individual PSM can be changed, probably promoted by the beneficial external practices or environmental influence [27]. Recently, some relevant empirical researches have confirmed that the leadership style is a powerful element of the influence on subordinate PSM [28, 29]. For example, Bellé (2014) emphasized the relationship between transformational leadership and PSM [30]. Jensen (2019) combined the effect of transactional leadership on PSM [31]. As one of the most frequently studied leadership styles in the public sector, servant leadership places considerable value on the care, support and help for the subordinates in order to improve their PSM [21]. It shows care and empathy for subordinates, and improve the quality of social relations with subordinates. When subordinates perceive the leader’s care, help and recognition, they will accept the values, goals and visions of the organization, and even perform altruistic service behaviors with more proactive service willingness to return the leader’s pay [32]. Meanwhile, domestic and foreign scholars have verified the vital function of PSM of people employed in public sectors on their extra-role proactive behaviors such as voice behaviors, organizing citizenship behaviors, innovative behaviors [33]. PSB is the specific extension of individual proactive behavior in the field of service [34], then likely to be influenced by PSM. Therefore, this study analyzed the mediating effect of PSM in the relation between servant leadership and PSB.

In addition, the influence of leadership on the subordinate motivation and behavior can also be affected by individual factors [35]. Though faced with the same leader, junior civil servants respond differently to its leadership style or manner due to their differentiated individual characteristics [6, 36]. Role identity emphasizes self-evaluation and self-adjusting of the individual’ s role, reflecting his or her cognitive intention of who one is and who one wants to be, which essentially means a self-adjusting characteristic in the process of individual’ interaction with the society [37]. In other words, the role identity level of junior civil servants could exert influence on one’ s cognition towards servant leadership [38]. Therefore, from the cognitive perspective, this study attempts to analyze how the individual differences of junior civil servants’ role identity influence the relation among servant leadership, PSM and PSB, which further clarifies the boundary of promotion strategy of the PSB of Chinese junior civil servants from the perspective of servant leadership.

**Fig. 1:**
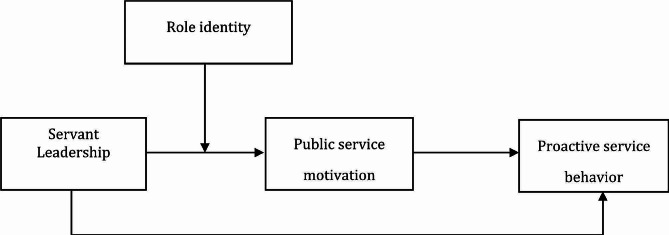
Research model

As the implementers of government policies, junior civil servants bear the responsibility of providing services to the public. Whether they can put themselves in the people’s shoes and show more active service consciousness directly reflects the government’s management ability and the realization of service-oriented government goals. Therefore, this study builds and verifies a moderated mediation model (as shown in Fig. 1), to reveal in what way and under what conditions servant leadership can more effectively influence PSB of junior civil servants in the context of government management. Specifically, this study not only confirms the effectiveness of servant leadership and PSB in the context of Chinese public management, but also provides a new perspective for research in these two fields and further clarifies the understanding of the mechanism of servant leadership’s influence on junior civil servants’ PSB. Besides, the mediating role of PSM clarifies its effectiveness in explaining the impact of servant leadership on subordinate behavior and, to a certain extent, opens the “black box” of the intrinsic effect of servant leadership on PSB to provide a more comprehensive understanding of this mechanism. In addition, the moderating role of role identity validates its applicability to new situations and reveals possible boundary conditions for the impact of servant leadership on PSB. Taken together, this study provides theoretical guidance for the government administration to exert positive influence more effectively of servant leadership on junior civil servants’ motivation and behavior, and also provide a reference on how to encourage junior civil servants to actively engage in proactive activities of serving the masses.

## Theoretical background and research hypotheses

### Social learning theory

Bandura (1973) first proposed the Social Learning Theory (SLT), which emphasizes that social learning is the most effective and important form of human learning. Much of human behavior is determined by observing and imitating the behavior of others and is reinforced by praise and acceptance [39]. The theory includes three core ideas: interactive decision theory, observational learning theory, and self-efficacy theory. SLT theory believes that individuals strengthen their cognitive experience by observing the behavior of others in the learning environment is the most important form of social learning, and emphasizes the important role of alternative experience, self-efficacy, and other factors in the emergence of individual behavior. In other words, SLT suggests that human behavior is determined by the functional relationship between the external environment, personal factors and the behavior itself [40].

In his research, Bandura emphasized the close relationship between social learning and individual behavior, and he believed that the complex behavior of individuals mainly originates from acquired [41]. Individuals acquire knowledge, skills, values, etc. through observation, imitation, and interaction in social learning processes, which are in turn influenced by the environment. The environments in which individuals live include family, school, work circle, etc., and these environments have an important impact on individuals’ social learning, shaping their behavioral patterns, cognitive structures, and social adaptability. Especially in the work environment, leaders, as an important external factor, their leadership styles shape the culture and work atmosphere within the organization, thus powerfully influencing employees’ behavior and other aspects [42].

Therefore, Bandura’s Social Learning Theory explains the law of individual behavior and provides theoretical support for the study of exploring the mechanism of the influence of servant leadership on the proactive service behavior of junior civil servants. Servant leadership, as a leadership style that emphasizes employee-centeredness and concern for their needs and development in the relationship with employees, can influence subordinates’ social learning process, which in turn promotes subordinates’ performance of proactive service behaviors [43]. Therefore, this study applies SLT to explore and reveal how servant leadership influences subordinates’ proactive service behaviors in the process of social learning.

First, in SLT, factors such as the power, status, ability and image of the imitated object have an impact on the observer’s willingness to learn [54]. As the person with the highest power and status in the team, the leader has a direct and close relationship with the team members, and his or her behavior is likely to be a role model for the team members. Second, servant leadership is a people-centered leadership style that prioritizes others over self-interest [44]. It is worth noting that there are some similarities between servant-leadership and the professional requirements of civil servants to serve the people. When servant-leaders promote the development and success of their team members by assisting and supporting others and providing resources, guidance, and motivation to meet their needs, team members are likely to realize the importance of offering help to others in order to achieve their personal and team goals and internalize this sense of service. They will try to apply it to their own roles, with better understanding and performance of their duties, exerting the function of serving the people. This results in intrinsic motivation for public service and further proactive service behaviors, such as proactively providing information, sharing resources, and assisting in problem solving [32], and this internalization process reflects the observation and imitation learning mechanisms in SLT.

Role identity refers to an individual’s self-examination about a specific role, reflecting the individual’s perception and willingness to know who he or she is and who he or she wants to be, and is a process of self-regulation in the process of interaction between the individual and society [37]. According to SLT, individuals learn and internalize appropriate behavioral patterns by observing and imitating leaders’ behaviors [40]. In the mechanism of servant leadership’s influence on PSB, role identity plays a key role as an important moderating variable. Individuals’ strong role identity may make it easier for them to accept and internalize servant leadership’s sense of service, thus motivating them to generate PSM and PSB. This is due to the fact that their strong role identity as civil servants will make them perceive the leader’s behavior as an appropriate and credible model to apply in their work, which motivates them to actively emulate and devote themselves to serving the people. However, negative role identity may diminish the impact of servant leadership because those may have difficulty connecting it to their work beliefs of serving the people, thus diminishing the overall impact effect.

### Servant leadership and PSB

To build a service-oriented government that satisfies the people needs servant leadership [25, 44]. Since the founding of the People’ s Republic of China, numerous servant leaders have emerged in government departments like Kong Fansen and Jiao Yulu. Servant leaders always take“serving others”as their mission with the priority of others’ demands, wishes and benefits ahead of theirs in daily work practices, and volunteer to offer their subordinates better opportunities to develop, aimed to foster excellent servicer for the society [20, 45]. For the servant leaders, the most important thing is to serve others. That is to say, they view it as motive power into their leadership process, which stimulates the subordinates’ internal impetus such as positive work moods, psychology, spirit and finally benefits the whole organization [46]. Numerous studies have shown that servant leadership can engender a positive impact on their subordinates, including enhancing helping behaviors, proactive behaviors, organizational citizenship behaviors, voice behaviors [47–50, 51]. According to SLT, because of their status, position and power at the center of the organization, servant leaders easily become the role model for organization members to observe and learn from [54]. In particular, civil servants have service-centered job requirements. For the leaders in the government departments, it is their service-oriented and altruistic leadership demonstrated before their subordinates that generates attention, learning and imitation of junior civil servants, which inspires them to assume their professional responsibility of “serving the people” in depth [55], and urges them to perform better and more proactively in policy implementation and service delivery. It means the positive influence of servant leadership on PSB of junior civil servants. Therefore, this study proposes the following research hypothesis based on this.

Hypothesis 1: Servant leadership positively affects PSB of junior civil servants.

### The mediating role of PSM

PSM has been a hot topic in the field of public administration since the new century [27, 56]. In essence, PSM emphasizes individual’s predisposition to respond to motives grounded primarily or uniquely in public institutions [26]. It is a self-positioning and internal motivation based on altruism, including but not limited to attraction of public organization members to policy making, commitment to the public interest, compassion and self-sacrifice [57]. As what Public Management School emphasizes, PSM focuses more on the altruistic behavioral motivations of public organizations or sectors, achieving a leap in application scope and active content compared to self-interested motivations of traditional private organizations or enterprises [32]. Many pervious researches have verified that leadership style or behavior is an important factor affecting individual PSM [21, 27, 56–59]. From the perspective of SLT, compared with other leadership styles, the qualities of servant leadership are the most compatible with the essence of PSM, with servant leaders regarded by their subordinates as having the qualities of serving the public, empathy, self-sacrifice, and other role models qualities, which could elicit learning and imitation from their subordinates to safeguard the public interest. Servant leadership is altruistic in their approach to service and contributes to the formation of a “service climate” in the public sector. This particularly affects subordinates’ perceptions of emotion, morality, and responsibility [33], suggesting that subordinates are more likely to want to serve the public interest under the influence of it. That is, servant leadership, which takes serving others as its orientation is beneficial to the enthusiasm and responsibility of members to contribute to public interest, then improving their PSM [21, 59]. Therefore, this study proposes the following research hypothesis.

Hypothesis 2: Servant leadership positively affects PSM of junior civil servants.

Existing relevant studies show that as other psychological processes [60], PSM of public organization members could be stimulated by the environment where they work and exert significant influence on individual working attitude, behavior and performance [32]. In other words, PSM is the important bridge to connect external environment elements and individuals’ own behaviors [31]. According to SLT, human behavior is determined by the functional relationship between the external environment, personal factors and the behavior itself. Specifically, junior civil servants with high PSM stimulated by servant leadership tend to have stronger will to serve the masses and society, higher identity level of government working purpose to serve the people and more self-awareness to devote to constructing servant government, then more PSB occur [52–53, 61]. Besides, on account of the nature and positioning of public sectors, servant leadership usually generates junior civil servants’ PSM through organizing and participating in activities of serving the people by their own [21, 59]. It is also consistent with the desire and inclination of junior civil servants to participate in public service, help others and serve society [26], which stimulates them to internalize the work purpose of serving the people and transform it into their actual work goals and contributes to fostering more active civil servants [62]. Based on above, this study argues that for the junior civil servants themselves, PSM can effectively transmit the positive influence of servant leadership as a situational factor, which in turn has a positive effect on their PSB. Therefore, this study proposes the following research hypothesis.

Hypothesis 3: PSM of junior civil servants mediates the positive effect of servant leadership on their PSB.

### The moderating role of role identity

It is widely known that the different roles that individuals play in different environments are the basis for their self-concept formation and are key factors in guiding their behavioral choices [63]. Role identity stems from two main sources: feedback about the self from social relations and associated self-views [64]. In other previous studies, altruistic motivation could also have a positive effect on role identity [65]. From the perspective of feedback from social relations, as the providers of employees’ work resources and cooperators in the daily work process, superiors’ evaluation and the quality of the relationship between superiors and subordinates play a decisive role in the choice of employees’ behaviors, which play an important role that drives employees to have positive role identity [66]. SLT states that individuals learn through observation to gain experience, increase their self-efficacy and psychological responsibility, and become more confident and motivated to complete an activity. Essentially, role identity is individual perception and attitude towards their organizational role, status and identity, which could moderate the interactive process among them and external contextual factors, further influencing individual motivation and attitude [38, 67]. Individuals with high role identity could timely catch the information from the outside, usually regulating their behavioral motivation according to external expectation to satisfy the needs of verifying and maintaining their role identity [68]. That is, when individuals have a strong sense of identification with their social role, they are more likely to be positively influenced and motivated by servant leadership, which in turn leads to PSM. Therefore, this study argues that the role identity of junior civil servants could moderate the influence of servant leadership on their PSM.

Role identity motivates role performances because enactment of relevant roles fulfills a critical need for self-verification [69] and allows relevant others to identify and categorize an individual [70]. The higher an individual’s role identity, the higher the probability that the individual’s behavior will be consistent with that identity [71]. Specifically, junior civil servants with high role identity always have explicit awareness in role definition of serving the people [72], which means more actions corresponding with the status of “providing service to the people proactively” and more sensitivity to service-oriented expectation [73]. When they are faced with servant leadership, their strong role identity as civil servants will make them easier to perceive the leader’s altruistic behavior and connect it to their work beliefs as an appropriate and credible model to apply in their work, which motivates them to actively emulate and be more willing to devote themselves to serving the people. On the contrary, the ones with low role identity tend not to adjust their perceptions and behaviors based on external expectations [74], which leads to low sensitivity to servant leadership and behaviors and difficulty of understanding the expectation from their leaders to serve others and of improving PSM. Therefore, this study proposes the following research hypothesis.

Hypothesis 4: Role identity of junior civil servants moderates the positive effect of servant leadership on their PSM.

Based on the above discussion of the mediating role of PSM and the moderating role of role identity, this study argues that the role identity further moderates the mediating role of PSM in the relationship between servant leadership and their PSB. Specifically, when junior civil servants have a higher degree of identification with their role of serving the people, servant leadership has a stronger motivational effect on PSM, thus increasing PSB of junior civil servants to a higher degree. However, when junior civil servants have a lower degree of identification with their role, the effect of servant leadership on their PSM is less positive, and the effect of their PSB is less transmitted through PSM. Therefore, this study proposes the following research hypothesis.

Hypothesis 5: Role identity of junior civil servants moderates the mediating role of PSM in the relation between servant leadership and it.

## Methods

### Procedure and participants

To test these hypotheses and the entire theoretical model, this study conducted questionnaire surveys among 600 junior civil servants from grassroots communities, township governments and sub-district offices in Shandong Province, China, which has the second largest population in China and the largest number of applicants for civil servants. We connected with people in charge of departmental administrations at first, and through the lists of public employee IDs, 600 civil servants were randomly selected. During data collection, the promise that the individual information would be only used for academic research and kept strictly confidential. Meanwhile, each participant was a given small gift to increase their interest and engagement. Furthermore, to improve data quality and avoid common method bias, along with previous research [75–77], this study collected the sample data at one-month intervals to decrease common method bias. A two-stage questionnaire survey was conducted. In the first stage, subordinates were asked to report their perceptions of servant leadership, role identity, and their demographic information. A total of 556 participants responded to this stage. In the second stage, subordinates were invited to complete a questionnaire on their PSM and PSB and about 543 questionnaires were received. After removing incomplete, ambiguous questionnaires and samples with obvious response patterns, a total of 416 sets of final sample data were obtained, yielding an effective rate of 69.3%. Specifically, participants were asked to fill out a questionnaire with items related to servant leadership, role identity, and their demographic information. After one month, they were invited to complete a questionnaire on their PSM and PSB. A total of 556 valid questionnaires were returned for the first survey and 543 for the second survey. After removing invalid questionnaires with incomplete information, obvious response patterns, and unmatched responses of the two surveys, a total of 416 sets of final sample data were obtained in the current study, yielding an effective rate of 69.3%. Among them, 248 were male (59.6%) and 128 were female (40.4%). In terms of the age distribution, 318 (76.4%) were young people aged 35 and below, and only 4 (9.6%) were people aged 55 and above. In terms of education background, 44 people (10.6%) graduate from high school and below, 66 people (15.9%) junior college, 235 people (56.5%) with undergraduate studies, 67 people (16.1%) with postgraduate education and 4 people (1.0%) with doctoral education. In terms of working experience, 116 people (27.9%) work for one year or less, 104 (25.0%) for one to three years, 65 (12.7%) for three to five years, 53 (12.7%) for five to ten years, 78 (18.8%) for more than ten years.

### Measures

The original scales of main variables involved in this study were all written in English, which need to be translated into Chinese. This study therefore firstly invited two educational management scholars proficient in both English and Chinese translated all items from English to Chinese. Then, another bilingual professor, who had not seen the original English version, translated the Chinese version back into English. We also asked this professor to comment on any ambiguously worded items, and she did not suggest any noteworthy changes. In addition, to further improve the accuracy of the translation and avoid cultural bias, this study also conducted a small-scale pretest to check the Chinese-English translation and check the meaning equivalence of all items. And all the response format was a 5-point Likert scale ranging from 1 = “strongly disagree” to 5 = “strongly agree”.

#### Servant leadership

The 7-item scale modified by Liden and colleagues was used to measure servant leadership [78]. An example item is " Even with personal issues, I usually seek help from my supervisor”. In the current study, the Cronbach’ s alpha for scores from servant leadership was 0.836.

#### Role identity

The 6-item scale developed by Smidts, Pruyn and Van Riel were applied to measure role identity [79]. An example item is " I have a strong sense of identity with my job as a civil servant”. In the current study, the Cronbach’ s alpha for scores from role identity was 0.874.

#### PSM

The 8-item scale developed and validated by Kim was adopted to assess PSM [80]. Though Chinese administrative divisions are mainly divided into three levels of organizational units, provinces, cities, counties and below, the civil servants in different departments follow the general framework of the national civil service system, which can conform to the dimensions of the refined PSM construct. Hence, the PSM scale was adopted. An example item is " I am willing to make my efforts for the public welfare “. In the current study, the Cronbach’ s alpha for scores from PSM was 0.821.

#### PSB

The 4-item scale developed and verified by Parker, Williams and Turner were introduced to rate PSB of junior civil servants [81]. PSB scale measures the ability of “universality” and common proactive work behavior. An example item is " I often find opportunity to improve the process and method in daily work “. In the current study, the Cronbach’ s alpha for scores from PSB was 0.866.

### Analysis strategy

This study used SPSS 22.0 software, Amos 22.0 software and SPSS PROCESS macro program to test the data. The specific process is as follows: First, internal consistency tests, exploratory factor analyses and confirmatory factor analyses are used to test the reliability and validity of core variables. Second, the Harman’s single-factor test is used to test the common method bias of this study. Third, descriptive statistics and correlations analyses are computed to preliminary test the relationships among core variables. Finally, hierarchical regression analyses are implied to check the direct effect of servant leadership on PSB and PSM, the mediating effect of PSM, and the moderating effect of role identity in the relationship between servant leadership and PSM. In addition, the PROCESS macro (Model 7) is introduced to test whether role identity moderates the mediating effect of PSM in the relationship between servant leadership and PSB.

## Results

### Reliability and validity verification

This study first performs a preliminary analysis to ensure that there is no violation of linearity or homoscedasticity. Specific test results show that the variance inflation factors (VIF) are all lower than 10, and the Durbin-Watson values fell within the acceptable range of 1.8-2.0 (a value close to 2), which means there is probably no autocorrelation problem in our sample data. Besides, the Cronbach’s alpha of servant leadership, role identity, PSM and PSB are 0.836, 0.874, 0.821 and 0.866, which indicating high reliability. Therefore, the measurement items used in this research have relatively high levels of internal consistency.

In addition, this study uses principal component analysis for factor extraction and exploratory factor analysis using variance maximum rotation to verify the validity among variables. The results are presented in Table 1, which indicate that the factor loading values of all items are 0.7 or higher (0.713 ∼ 0.886). The Bartlett’s test statistic for sphericity, which verifies whether the correlation between variables is 0, is 4910.010 (df = 300, *p* = 0.000), and the KMO value measuring the adequacy of our final sample is 0.911, which is close to 1. Therefore, this study believes that these four variables can be clearly distinguished and suitable for factor analysis.

**Table 1:** Results of exploratory factor analyses

Factor	Measure	1	2	3	4
Servant leadership (Cronbach’ s alpha = 0.836)	SL 1	0.813			
	SL 2	0.799			
	SL 3	0.809			
	SL 4	0.786			
	SL 5	0.775			
	SL 6	0.847			
	SL 7	0.810			
Role identity (Cronbach’ s alpha = 0.874)	RI 1		0.797		
	RI 2		0.796		
	RI 3		0.824		
	RI 4		0.835		
	RI 5		0.799		
	RI 6		0.869		
PSM (Cronbach’ s alpha = 0.821)	PSM 1			0.870	
	PSM 2			0.713	
	PSM 3			0.797	
	PSM 4			0.807	
	PSM 5			0.805	
	PSM 6			0.830	
	PSM 7			0.879	
	PSM 8			0.841	
PSB (Cronbach’ s alpha = 0.866)	PSB 1				0.861
	PSB 2				0.815
	PSB 3				0.866
	PSB 4				0.886
Eigenvalue		8.424	2.289	1.965	1.792
% of variance		33.696	9.154	7.859	7.167
% of cumulative		33.696	42.851	50.710	57.877
KMO = 0.911, Bartlett (χ^2^ = 4910.010, *df* = 300, *p* = 0.000)					

This study further conducts confirmatory factor analyses to test factor structure and construct validity of our proposed model. The results are presented in Table 2. It can be observed that the four-factor measurement model (with servant leadership, role identity, PSM and PSB as four independent factors) exhibits a better fit to the data (χ2/df = 2.280 <3, CFI = 0.930 >0.9, TLI = 0.919>0.9, RMSEA = 0.056 <0.8, SRMR = 0.077 <0.8) than the other three measurement models, which demonstrates that the respondents have a good discriminant validity in the current study.

**Table 2:** Results of Confirmatory Factor Analyses

Model	Factor	χ^2^/df	RMSEA	CFI	TLI	SRMR
Four-factor model	SL, PSM, RI, PSB	2.280	0.056	0.930	0.919	0.077
Three-factor model	SL, PSM + RI, PSB	3.184	0.091	0.880	0.861	0.091
Two-factor model	SL + PSM + RI, PSB	5.211	0.101	0.766	0.732	0.128
Single-factor model	SL + PSM + RI + PSB	6.565	0.116	0.688	0.646	0.143

*Note*: *N* = 416; SL = servant leadership; RI = role identity; Ideal model-fit indicators are: χ^2^/*df* < 3, CFI > 0.9, TLI > 0.9, RMSEA < 0.08, SRMR < 0.08.

### Common method bias analysis

To reduce the influence of common method bias on the result, this study clearly stated in the instruction section of the questionnaire that this survey does not involve any personal privacy with no signature required. Moreover, all the question options are no good or bad and nothing personal information will be leaked to ensure that the junior civil servants involved in the investigation could answer safely and honestly. Harman’s single-factor test was used to examine the data obtained from the survey [82], and the result showed that one factor extracted and explained only for 33.696% of the variance, which was less than the suggested criterion of 50%. Therefore, the sample data obtained in this study does not have the problem of excessive interpretation of a single factor. In addition, the results of confirmatory factor analysis (CFA) combining all the indexes also exhibit a poor fit to the data (χ2/*df* = 6.565, RMSEA = 0.116, CFI = 0.688, TLI = 0.646, RMR = 0.143), with fitting index well below evaluating index [83]. Therefore, the common method bias is not a major issue and the measurement scales used in the current study have good reliability and validity.

### Descriptive statistics and correlation analysis

Table 3 shows the means, standard deviations and intercorrelations for the main variables. As predicted in this study, servant leadership is positively correlated not only with junior civil servants’ PSM (*r* = 0.536, *p* < 0.01), but also PSB (*r* = 0.306, *p* < 0.01). Besides, PSM is also significantly and positively related with their PSB (*r* = 0.392, *p* < 0.01). Moreover, role identity is also significantly and positively related with servant leadership (*r* = 0.493, *p* < 0.01), PSM (*r* = 0.626, *p* < 0.01) and PSB (*r* = 0.393, *p* < 0.01). Taken together, these results can provide preliminary evidences for the relationship among the main variables and foundation for the subsequent regression analysis and model testing in the current study.

**Table 3:** Descriptive statistics and correlation coefficients of variables

	1	2	3	4	5	6	7	8
1.Gender								
2.Age	-0.119*							
3.Education	-0.014	0.157**						
4. Year	-0.068	0.372**	0.185**					
5.SL	-0.007	-0.028	0.051	0.104*				
6.PSM	0.082	-0.006	0.004	0.155**	0.536**			
7.RI	-0.009	0.012	-0.018	0.109*	0.493**	0.626**		
8.PSB	0.121*	-0.014	0.182**	0.052	0.306**	0.393**	0.392**	
Mean	1.596	2.022	2.810	2.695	2.566	2.501	2.613	3.128
SD	0.491	0.990	0.864	1.468	0.785	0.801	0.916	1.075

*Note*: *N* = 416; *SD* = Standard Deviation; SL = servant leadership; RI = role identity; * *p* < 0.05, ** *p* < 0.01

### Hypotheses testing

This study firstly uses SPSS 22.0 to conduct hierarchical regression analysis to test hypotheses 1–4, including direct effects, mediating effects and moderating effects among the main variables. The results are presented in Table 4. Next, the PROCESS Macro is used to verify the mediated mediation effect, that is, Hypothesis 5 and the whole theoretical model.

For the direct effect of servant leadership on PSM and PSB, as shown in Model 1 and Model 2, servant leadership significantly has a positive influence on PSM (*β* = 0.526, t = 12.731, *p* < 0.001). Moreover, according to the results of Model 4 and Model 5, servant leadership also significantly has a positive effect on PSB of junior civil servants (*β* = 0.297, t = 6.558, *p* < 0.001). Therefore, Hypothesis 1 and Hypothesis 2 are supported.

**Table 4:** Results of Hierarchical Regression Analyses

Variables	PSM				PSB			
	Model 1	Model 2	Model 3		Model 4	Model 5	Model 6	Model 7
Gender	0.092	0.092*	0.120^**^		0.127	0.126	0.098	0.138^**^
Grade	0.030	0.022	0.028		0.031	0.026	0.019	0.025
Major	-0.048	-0.052	-0.037		0.176^***^	0.174^***^	0.190^***^	0.187^***^
Tenure year	0.158^***^	0.107^**^	0.079^*^		0.043	0.006	-0.031	-0.019
SL		0.526^***^	0.241^***^			0.297^***^	0.139^**^	0.118^*^
PSM							0.307^***^	
RI			0.403^***^					0.301^***^
SL × IR			0.236^***^					0.099^*^
R^2^	0.036	0.308	0.518		0.051	0.137	0.203	0.230
ΔR^2^		0.272	0.210			0.086	0.065	0.179
F	3.870**	36.467^***^	62.649^***^		5.501^***^	13.058^***^	17.335^***^	17.366^***^

*Note*: *N* = 416; SL = servant leadership; RI = role identity; * *p* < 0.05, ** *p* < 0.01, *** *p* < 0.001

Then, for the mediating effect of PSM, this study followed Baron and Kenny’ procedures to verify the mediating effect [84]. On the basis of Model 4 and Model 5, PSM was put into the regression equation and Model 6 was obtained. The result shows PSM of junior civil servants has a significant positive effect on their PSB (*β* = 0.307, t = 5.851, *p* < 0.001) and servant leadership still has a significant positive effect on PSB (*β* = 0.139, t = 2.659, *p* < 0.01). These indicate that PSM partly mediates the positive influence of servant leadership on their PSB. Therefore, Hypothesis 3 is supported.

**Fig. 2:**
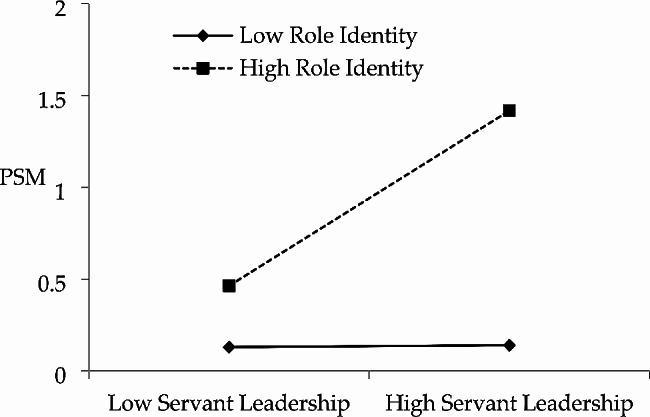
Moderating effect of role identity in the relationship between servant leadership and PSM

Furthermore, for the moderating effect of role identity, the results of Model 2 and Model 3 show that the interaction term has a significant positive effect on junior civil servants’ PSM (*β* = 0.236, t = 6.150, *p*<0.001), indicating that role identity plays a significant positive moderating role in the influence of servant leadership on PSM. This indicates that role identity plays a significant positive moderating role in the influence of servant leaders on the motivation of grassroots civil servants. To further demonstrate the moderating effect, Fig. 2 shows the moderating effect of role identity in the relationship between servant leadership and PSM with one standard deviation positive and negative. As can be seen from the graph, servant leadership significantly enhances PSM of junior civil servants when they have high role identity, otherwise the facilitative effect of servant leadership on their PSM is weaker when role identity is low. Therefore, Hypothesis 4 was supported.

Finally, this study applied Bootstrap methods and learned from scholars Hayes and Preacher in virtue of PROCESS macros [85]. To be specific, this study bootstrapped with 5000 in order to generate bias-corrected confidence intervals of yield 95%. The results are presented in Table 5. The result shows that the whole model test index with PSM as the mediating role and role identity as the moderating role is 0.079, 95% CI = [0.036, 0.132], which excluding zero. This suggests that role identity moderates the mediating role between PSM and PSB. Specifically, when the mean value of role identity is subtracted by one standard deviation, 95% CI of the overall moderated mediating effect is [-0.018, 0.080] containing zero; while when the mean value of role identity is increased by one standard deviation, 95% CI of the overall moderated mediating effect is [0.103, 0.262] excluding zero. It can be inferred that the mediating effect of PSM in the relationship between servant leadership and subordinate PSB becomes more significant with the increase of role identity of junior civil servants. Take together, Hypothesis 5 was well supported.

**Table 5:** Results of Bootstrap Analyses

Conditional indirect effect	Effect	SE	95%	
			LLCI	ULCI
M– 1 SD	0.029	0.025	-0.018	0.080
M	0.102	0.252	0.060	0.161
M + 1 SD	0.175	0.041	0.103	0.262
**Index of moderated mediation**				
PSM	0.079	0.025	0.036	0.133

*Note*: *N* = 416; Bootstrap sample size = 5,000. LL = lower limit; CI = confidence interval; UL = upper limit

## Discussion

From the perspective of servant leadership, this study took Chinese junior civil servants as objects with empirical method to analyze the promotion strategy for their PSB and verify the mediating role of PSM and moderating role of role identity. The results show that servant leadership efficiently improves PSB of junior civil servants. Besides, their PSM partly mediates the positive effect of servant leadership on PSB. In addition, role identity not only moderates the positive effect of servant leadership on PSM, but its mediating effect on PSB of junior civil servants. In other words, the higher role identity of junior civil servants, the more significant positive effect of servant leadership on public service motivation and the more obvious the mediating role of PSM.

### Theoretical implications

From the perspective of strategy of improving PSB, this study confirms the positive effect of servant leadership on junior civil servants’ PSM, then generating more PSB, which provides theoretical basis and evidence support [34, 37, 86]. Since the concept of PSB was proposed, relevant researches mainly have focused on accommodation and food services, banking and other traditional service industries [9, 87]. This study introduced the concept into public management and discussed the promotion strategy for PSB of junior civil servants, which not only confirmed the validity of this concept in Chinese public management context [16], but broadened the research field of individual PSB [11, 15], successfully offering a new view for study of this field and further clarifying the understanding of influence mechanism of junior civil servants’ PSB [12, 16, 88].

Secondly, this study reveals the positive effect of servant leadership on PSM and PSB of junior civil servants, which answers the call for strengthening the research on the effect of servant leadership from the academic circle [20, 45, 48, 62] and enriches the relevant researches in China [25, 47]. With the core of service, the influence of servant leadership on subordinates such as motivation, attitude, behavior and performance have been confirmed by numerous studies not only in China but also abroad [48]. However, based on this, a few studies aimed to analyze the relationship between servant leadership and their subordinates in Chinese government management. This study took Chinese junior civil servants as research objects, confirmed the positive influence of servant leadership on public service motivation and behaviors, providing some support for researches on servant leadership and construction of the theoretical system of servant leadership in Chinese context [6, 50, 55], which also enriching the researches on servant leadership in the academic circle and the influence path from the view of individual aspect demonstrates applicability in the whole world.

Thirdly, in the context of Public Administration, the mediating role of PSM suggests the influence mechanism of servant leadership on PSB. With the in-depth studies about public administration, how to effectively increase PSM of civil servants has become a hot research topic [29, 62]. This study verifies the positive effect of public servant leadership and the mediating role of PSM [27]. That is to say, PSM can effectively mediate the influence of servant leadership on PSB of junior civil servants [32]. It is clear that the validity of PSM to explain the influence of servant leadership on subordinates’ behaviors has been verified [26, 60, 61], expanding application scope and providing some theoretical interpretation of the concept [27], which opens the “black box” of the intrinsic effect of servant leadership on PSB to a certain extent for understanding the mechanism more comprehensively [56, 62].

Finally, the moderating effect of role identity in the relation between public service and PSB was analyzed in this study, enriching the relevant researches about individual role identity in the academic circle [38, 89]. Previous studies suggest that this novel concept can effectively moderate the interaction process between individuals and external contextual factors and further exert impact on individuals’ motivation and behaviors [63, 68, 90]. In this study, role identity was introduced into the field of Public Management, expanding its theoretical interpretation to some extent and verifying its applicability in a new context. In addition, this finding also shows the higher degree of role identity, the more significant effect of servant leadership on PSM, and the more significant mediating effect of PSM on servant leadership and PSB [37, 91], which reveals the possible boundary conditions for the impact of servant leadership on PSB.

### Managerial implications

To further improve PSB of junior civil servants for constructing the service-oriented government, for one thing, it is essential that leaders are encouraged to change their roles actively and fulfill servant leadership throughout daily practice, giving the priority of others’ demands, wishes and benefits, taking the lead to offer their subordinates high-quality service and then engendering more PSB to serve the masses. For another, government management departments are supposed to develop activities to enhance service awareness and special service skills of leaders, truly achieving“servant leadership”, which plays a stimulative role of guiding junior civil servants’ PSB as to build a real service-oriented government.

Moreover, due to its special role to connect servant leadership and PSB, government management departments must realize the significance of PSM and merge it into the system for the selection, evaluation and promotion of civil servants, prioritizing who with high public service motivation in case of similar conditions. Meanwhile, leaders in government management departments are also encouraged to hold philosophy of “leaders serve subordinates while subordinates serve the people”, treat their subordinates through servant leadership and attach more importance to their own words and deeds in daily work as to demonstrate a serving-others working atmosphere. Besides, encouraging junior civil servants to truly be involved in policy making relevant to public service by giving adequate trust and support can also fundamentally promote their PSM.

In the end, role identity strengthens the promotion influence of servant leadership both on PSM and PSB of junior civil servants, which means the necessity of cultivating their job role identity conducive to serve the masses. To be specific, daily ideological and political education should be emphasized, and then create immersive cultural atmosphere of serving the people by combining the concentrated display along with daily propagandizing. For example, public spaces such as canteen, corridor or elevator can be utilized to put up relevant posters, pictures or slogans, or even broadcast the deeds of typical service leaders as to imbibe junior civil servants with the serving-others philosophy and help them establish working goal and vision of serving the people, truly making them realize the importance of role identity towards service-oriented government construction and increase it consciously.

### Limitations and recommendations

There are still some limitations in the current research, which can also provide some inspiration for future researches. Firstly, limited to objective condition, the sample data of this study was collected only from one city in China, which means the limited sample scope and sources [47, 92, 93]. At the same time, the sample data all came from self-evaluation of junior civil servants, leading to common method bias to some extent, which provides some inspiration for future researches to collect more high-quality sample by enlarging sample range or using multi-source method. Secondly, though two-stage approach was used to collect sample, the relationship among servant leadership, PSM and PSB of junior civil servants has not been entirely confirmed. Hence, future researches could adopt different methods like interview or experiment to verify the conclusions. Thirdly, except the mediating role of PSM and moderating effect of role identity, other variables could also play a part, thus, future researches could go further by incorporating other mediating or moderating variables from different perspectives and theories to explore the promotion strategy of junior civil servants’ PSB. Finally, by systematically reviewing the research literature on servant leadership and PSB, the main findings and research limitations of existing empirical studies are found in this study, and the definition and connotation of each variable in the context of organizational management and the main theoretical basis of the impact of servant leadership on PSB are clarified. However, the current reference of this study still has certain limitations, and future studies can further break through the perspective and enhance the explanatory power of the theory.

## Conclusions

In conclusion, this study explored the promoting effect of servant leadership on PSB of Chinese junior civil servants. The mediating role of PSM and the moderating role of role identity in the above facilitative relationship were also discussed in detail. The empirical test results showed that servant leadership can positively affect PSM of junior civil servants, and then promote their PSB. Moreover, role identity plays a positive moderating role in the above relationships, that is, the higher the level of role identity of junior civil servants, the more likely they will have a high level of PSM when they are influenced by servant leadership, and thus generate more PSB. Despite the limitations, this study hopes that our findings will prompt other researchers to further improve their scholarly understanding of servant leadership and proactive service behaviors in public organizations.

## Data Availability

Data supporting the results of this study are available from Y.Y., the corresponding author. The data cannot be made public because it contains information that could compromise the privacy of study participants.
